# The **α**_1_ *AT* and *TIMP-1* Gene Polymorphism in the Development of Asthma

**DOI:** 10.1155/2012/968267

**Published:** 2012-11-21

**Authors:** Manish Kumar, D. P. Bhadoria, Koushik Dutta, Mitesh Kumar F., Bharat Singh, Seema Singh, Anil K. Chhillar, D. Behera, G. L. Sharma

**Affiliations:** ^1^CSIR-Institute of Genomics and Integrative Biology, University Campus, Mall Road, Delhi 110007, India; ^2^Centre for Biotechnology, Maharshi Dayanand University, Rohtak, Haryana 124001, India; ^3^Maulana Azad Medical College and Lok Nayak Jai Prakash Narayan Hospital, New Delhi 110002, India; ^4^Lala Ram Sarup Institute of Tuberculosis and Respiratory Diseases, New Delhi 110030, India

## Abstract

Asthma has been an inflammatory disorder accompanied by tissue remodeling and protease-antiprotease imbalance in lungs. The SNPs of *alpha-1 antitrypsin* (**α**_1_ *AT*) and *tissue inhibitor of metalloproteinase-1* (*TIMP-1*) genes were studied for their association with asthma. Genotyping of **α**_1_ *AT* and *TIMP-1* genes was performed in 202 asthmatics and 204 controls. Serum levels of **α**_1_AT, TIMP-1 and cytokines were estimated to find if the interplay between genotypes and cellular biomarkers determines the pathogenesis of asthma. The analysis of results showed significantly low level of **α**_1_AT in the serum of asthmatics as compared to controls (*P* = 0.001), whereas cytokines were elevated in patients. No significant difference was observed in the concentration of TIMP-1 in patients and controls. Genotyping led to the identification of 3 SNPs (Val213Ala, Glu363Lys, and Glu376Asp) in **α**_1_ *AT* gene. The novel SNP Glu363Lys of **α**_1_ *AT* was found to be associated with asthma (*P* = 0.001). The analysis of *TIMP-1* gene showed the occurrence of seven SNPs, including a novel intronic SNP at base G3774A. The allele frequency of G3774A and Phe124Phe was significantly higher in asthmatics as compared to controls. Thus, the SNP Glu363Lys of **α**_1_ *AT* and G3774A and Phe124Phe of *TIMP-1* could be important genetic markers for use in better management of the disease.

## 1. Introduction

 Asthma pathophysiology involves extensive remodeling of lung tissue structure, and the protease-antiprotease imbalance has been considered to play a significant role in the pathogenesis of the disease [1]. The neutrophils and alveolar macrophages (AM) synthesize neutrophil elastase (NE) and matrix metallo proteinase (MMP), respectively, which have been the prominent proteolytic enzymes released during the inflammatory events [2]. The NE is a protease which destroys major structural proteins of the alveolar wall. Since the expression and the activity of NE are required to be kept under check for the maintenance of the lung structure and integrity, an antiprotease alpha-1 antitrypsin (*α*_1_AT) is produced in the body. The *α*_1_AT, primarily synthesized by hepatocytes, attenuates NE in the lungs [3]. The MMPs have been reported to play an important role in regulating a variety of cellular processes including proliferation, differentiation, migration (adhesion/dispersion), angiogenesis, host defense, and apoptosis [4, 5]. The activity of MMP-9, a major MMP present in lung tissues, is kept under tight control by the tissue inhibitor of metalloproteinase-1 (TIMP-1) [6]. It has been reported that the balance between MMP-9 and TIMP-1 gets disturbed in the airways of asthmatics. The increased effective concentration of MMP-9 has been shown to correlate with a fall in FEV1 [7].

 The link between asthma and *α*_1_AT deficiency has been demonstrated mainly in the heterozygote PIMS and PIMZ genotypes of **α**_1_ *AT* gene. The children with severe asthma had a greater frequency of Z heterozygote variants of **α**_1_ *AT* than the nonsteroid-dependent asthmatic and control population [8]. Asthma was found to be three-times more prevalent in PiMZ group as compared to the PiZZ group [9]. The studies from our laboratoty demonstrated PIM3 allele of **α**_1_ *AT* to be associated with COPD in patients recruited from northern plains of India [10, 11]. Since there has been significant overlap in the pathogenesis and symptomatology of asthma and COPD, we thought it is pertinent to look for possible role of **α**_1_ *AT* gene in asthma also. There is a lack of information on *TIMP-1* status in asthmatics and also its SNPs had not yet been studied in Indian population. Therefore, the current study was focused on investigating the role of **α**_1_ *AT* and *TIMP-1* at gene and protein levels and their interaction with inflammatory cytokines, in the development of asthma. 

## 2. Materials and Methods

### 2.1. Subjects

 Two hundred and two patients of asthma were recruited from the respiratory outpatient clinic of Lok Nayak Hospital, New Delhi, India, and 204 healthy individuals all unrelated North Indians were also enrolled as control subjects. Asthma was diagnosed on the basis of history, physical examination, and spirometric data, according to the Global Initiative for Asthma report and guidelines published in 2008, under the “Global Strategy for Asthma Management and Prevention.” The study protocol was approved by the Ethics Committee of Lok Nayak Hospital of Maulana Azad Medical College, New Delhi, India, and CSIR-Institute of Genomics and Integrative Biology, Delhi, India. Patients with fever, evidence of active respiratory tract infection, acute renal failure, active hepatitis at the time of visit or within previous four weeks, bronchiectasis, and tuberculosis indicated by chest X-ray and high resolution CT scan were excluded from the study. The subjects who received systemic corticosteroids in the previous four weeks were also excluded. Written informed consent was obtained from all subjects before participating in the study. The demographic details and smoking prevalence for all participants were assessed through a questionnaire. The body mass index (BMI) and smoking index (SI) were recorded for each subject. The blood was drawn from healthy individuals as well as the patients (age ≥13 years) who were diagnosed for asthma. 

### 2.2. Genotyping

The DNA was extracted from peripheral blood samples by the standard method [12] and a working DNA concentration of 50 ng/mL was used for genotyping experiments. The primers as described previously were synthesized and used for amplification of **α**_1_ *AT* and *TIMP-1* genes [11]. The PCR was carried out at optimized conditions [11] using a thermocycler (Gene Amp 9700, Applied Biosystems, Life Technologies, Singapore). The amplified PCR products were purified and subjected to sequencing using a 3730 sequencer (ABI Prism, USA). The SNPs were first identified by full length sequencing of PCR products of few subjects and subsequently large batches of samples were analysed for polymorphic genotypes by SNapshot Multiplex Kit (ABI Prism, USA) using SNapshot primers (Table 1). Individual nucleotide peaks were identified using the ABI-Prism GeneScan and Genotyper (ABI) to determine the genotypes.

### 2.3. Serum Biochemistry

 The concentration of *α*_1_AT, TIMP-1, total IgE, and cytokines in the serum were determined as described below.

The level of *α*_1_AT in the sera was measured as serum trypsin inhibitory capacity (STIC) by a colorimetric assay as per method described earlier [13]. The absorbance was measured using a spectrophotometer (Spectra Max 384 plus, Molecular Devices, USA) at 410 nm and the STIC was expressed as milligram of trypsin inhibited/mL of aserum sample. 

The level of TIMP-1 in serum samples of study subjects was determined by standard ELISA based microassay using a kit (DuoSet ELISA development system catalogue no. DY 970, R&D Systems, Inc., Minneapolis, MN, USA) as per method described earlier [11]. The absorbance in the wells of plate was recorded at 450 nm using a spectrophotometer (Spectra Max 384 plus, Molecular Devices, USA). The concentration of TIMP-1 was determined using a standard curve and expressed as ng/mL.

 Total serum IgE was determined by a sandwich ELISA using a commercially available kit (Bethyl laboratories, Inc. USA). The ELISA was performed according to the protocol provided by the manufacturer. The color in the wells was developed using 2, 2′-azino-bis 3 ethylbenzthiazoline-6-sulphonic acid, in the dark for 20 min and the reaction was stopped by adding 50 *μ*L of 2N H_2_SO_4_ to the wells. Absorbance of the plate was recorded at 405 nm. The level of total IgE was determined using the standard curve and expressed as IU/mL of the serum sample. 

The level of cytokines, namely, IL-1*β*, IL-4, IL-5, IL-6, IL-8, and IL-10 in serum samples of subjects was determined by standard ELISA based microassay using respective kits (DuoSet ELISA development system, R&D Systems, Inc., Minneapolis, MN, USA) as per the protocol provided by manufacturer (R&D Systems, Inc., Minneapolis, MN, USA). The concentrations of serum cytokines were determined using a separate standard curve for each cytokine and results were expressed as pg/mL of the serum sample. All the serum biochemistry parameters were analyzed in duplicate and experiments were repeated at least twice.

### 2.4. Statistical Analysis

Hardy-Weinberg equilibrium of alleles was assessed at the individual loci by goodness of fit test. The *χ* ^2^ test was performed for comparison of genotypes and allele frequencies for **α**_1_ *AT* and* TIMP-1* polymorphisms in the patients of asthma and control individuals. The Odds ratio (OR), 95% confidence interval (CI), and “*P*” values of genotyping data were adjusted for confounding factors such as age, gender, and SI. The biochemical parameters were expressed as mean ± SE. The difference between the groups was analyzed by *F*-test for equality of variance. In case of equal variance, Student's *t*-test was used and, for unequal variance, Welch test was employed. All tests were performed using softwares, SPSS 12 (SPSS Inc., Chicago, Illinois, USA) and EPIINFO 6 (Centers for Disease Control, Atlanta Georgia, USA). The analyses of various parameters using the above-mentioned statistical methods and softwares, the “*P*” value of ≤0.05 was considered to be statistically significant.

## 3. Results

**Figure 1:**
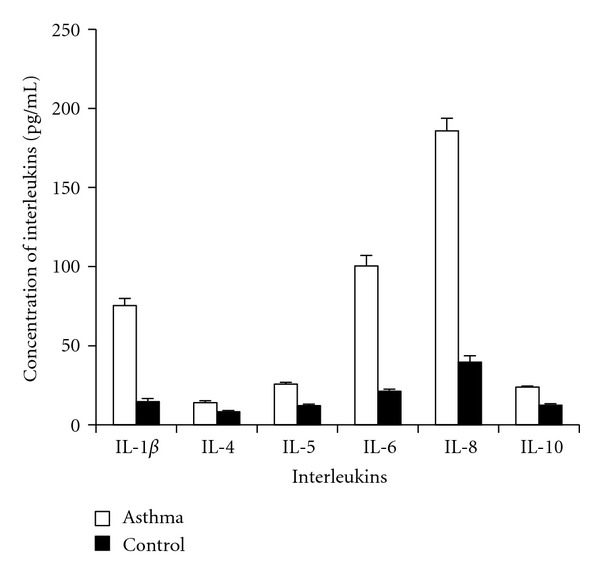
Serum levels of cytokines (mean ± SE) in asthma patients and control individuals.

The clinical, biochemical, and genotyping data obtained for all the asthmatics as well as control subjects were analyzed in the light of asthma prevalence and pathogenesis. The demographic and clinical characteristics of patients are provided in Table 2. The comparative analysis of total IgE in the sera samples of controls and asthma patients by ELISA demonstrated that there was a significant increase in total IgE in the serum of asthma patients than control individuals (*P* = 0.0001). The concentration of *α*_1_AT determined as STIC was found to be significantly (*P* = 0.001) decreased in the serum of asthmatics as compared to controls (Table 2). The concentration of TIMP-1 in the sera of patients of asthma was also less than that in controls, but the difference was not significant (Table 2). We observed an increase in the level of IL-1*β*, IL-4, IL-5, IL-6, IL-8, and IL-10 in asthma patients as compared to the controls (Figure 1).

The analysis of amplified gene sequences demonstrated three SNPs viz Val213Ala (T/C) in exon-III and Glu376Asp (A/C) also known as PIM3, and a novel SNP Glu363Lys (G/A) in exon-V of **α**_1_ *AT* gene, and seven SNPs (C/G rs5953060 in intron-IV, T/C rs4898 Phe124Phe in exon-V, G/A rs6609533 in intron-V, C/T Ile158Ile in exon-VI, and G/T rs2070584 as well as A/G rs6609534 in 3′ UTR and a novel G to A polymorphism at base position G3774A in the intron-IV) in *TIMP-1 *gene. The genotype frequencies for all SNPs of both **α**_1_ *AT* and *TIMP-1* genes were consistent with Hardy-Weinberg equilibrium. The genotype and allele frequency data for patients as well as control individuals was adjusted for confounding factors, that is, age, BMI, and SI and corrected “*P*” value was used, to compare the case with control.

**Figure 2:**
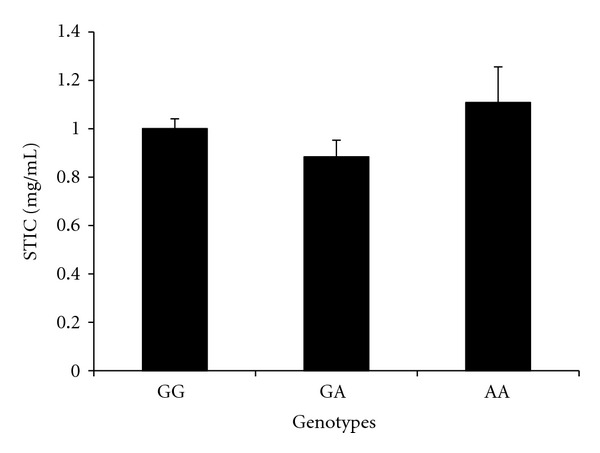
Serum trypsin inhibitory capacity (mean ± SE) in asthma patients having different genotypes of Glu363Lys of **α**_1_ *AT* GG, GA and AA are genotypes.

The analysis of sequencing data of **α**_1_ *AT* gene revealed that there was no significant difference in the distribution frequency of any of the three genotypes (TT, TC, and CC) of Val213Ala in controls and patients of asthma (Table 3). The Glu376Asp SNP (PIM3) in exon-V of **α**_1_ *AT* gene also did not show any association with asthma at the level of genotype or allele distribution (Tables 3 and 4). The genotypes GA and AA for the novel polymorphism Glu363Lys of **α**_1_ *AT* were found to be significantly associated with the prevalence of asthma (Table 3). The minor allele A was present in 9.80% controls and 20.54% patients of asthma and thus was considered to be associated with disease (Table 4). The SNP Glu363Lys did not show any association with **α**_1_ *AT* level in sera of asthmatics as patients of all three genotypes GG, GA and AA had similar levels of **α**_1_ *AT* (Figure 2).

The genotyping data for *TIMP-1* was segregated for male and female subjects (Table 3), as the gene remains located on the X-chromosome. The SNPs, rs5953060 in intron-IV, rs6609533 in intron-V and Ile158Ile in exon-VI of *TIMP-1* gene did not show any association with the disease in either sex. The SNPs rs2070584 as well as rs6609534 located in 3′ UTR, also were not found to be involved in development of asthma. However, the analysis of SNP Phe124Phe of *TIMP-1* gene showed the significant difference between homozygous TT and CC in females as well as in males (T/C) of asthma and control groups (*χ* ^2^ = 8.84, *P* = 0.002; *χ* ^2^ = 4.57, *P* = 0.03). The genotype frequency of heterozygous TC of Phe124Phe was not statistically different for asthmatic and control females (*χ* ^2^ = 1.83, *P* = 0.18). The minor allele C was present in 31.21% controls and 46.95% asthmatics in males and females together (Table 3). The allele distribution of this exonic SNP Phe124Phe was also found to be significantly different in asthma and control individuals (*P* = 0.02). This pattern of allelic distribution indicated a strong association of Phe124Phe SNP of *TIMP-1* gene with the prevalence and pathogenesis of asthma in Indian population. The TIMP-1 serum concentration in asthma patients with different genotypes (T, TT, TC, C and CC) of Phe124Phe did not differ significantly from each other (Figure 4), therefore, no interaction could be established between the antiprotease concentration and the silent SNP Phe124Phe in patients of asthma.

The genotype distribution for rs2070584 and rs6609534 did not show any association with asthma as evident from the results shown in Table 3.

**Figure 3:**
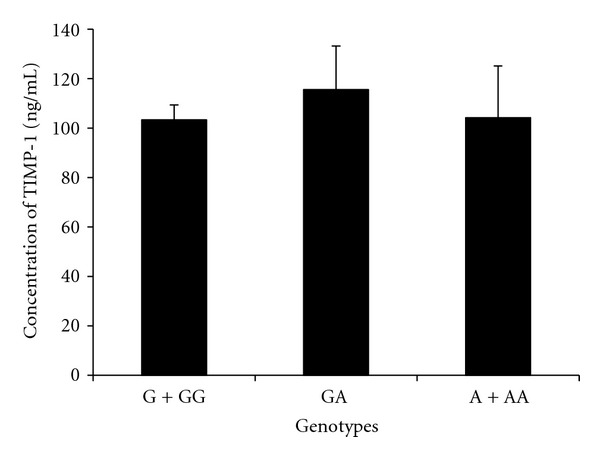
Concentration of TIMP-1 (mean ± SE) in the serum of male and female patients of asthma having different genotypes of Novel SNP 3774 of *TIMP-1* gene. GG (female) and G (male) are wild-type genotypes. GA, AA (female), and A (male) are mutant genotypes of *TIMP-1* gene.

The genotype distribution for novel SNP G3774A of *TIMP-1* gene was determined in subjects of both the groups. The heterozygous GA was significantly increased in asthmatic females as compared to controls (Table 3). The minor allele A was present in 3.91% controls and 14.79% asthmatics (Table 4). The allele frequency for the SNP G3774A was significantly different in asthma and control subjects (*P* = 0.008) indicating its strong link with the risk of development of asthma. The serum concentration of *TIMP-1* in asthmatics with different genotypes of the novel polymorphism G3774A was not found to be significantly different from each other (Figure 3).

## 4. Discussion 

 The present study investigated the status of *α*_1_AT and TIMP-1, the two major anti-protease molecules, in asthmatics. The possible interactions between the SNPs of these two genes (**α**_1_ *AT* and *TIMP-1*) and their association with asthma in Indian population were also explored. It was observed that the STIC in asthmatics was significantly lower than that in control individuals. The normal value of STIC in healthy individuals in different populations has been reported to be in the range from 1.0 to 2.1 mg of trypsin inhibited/mL of serum [14–16]. The earlier studies correlating the asthma features and *α*_1_AT levels have shown variable observations. A study conducted by Miravitlles et al. demonstrated that 22 out of 111 (19.8%) of asthmatics with a deficient phenotype of **α**_1_ *AT* gene had normal values of *α*_1_AT [17]. Also, no significant differences were found when **α**_1_ *AT* values were compared with each of the asthma patient groups classified according to severity criteria [17]. Eden et al. had reported that 38% of subjects, having asthma with PIZZ genotype, had very low levels of *α*_1_AT in their sera [9]. Aderele et al. [18] reported that the serum levels of *α*_1_AT in asthmatic children were significantly lower in controls (*P* < 0.02). We have observed a decreased *α*_1_AT activity in patients of asthma and these results are in agreement with other studies carried by various workers [9, 17, 18]. 

The concentration of TIMP-1 in the sera of patients of asthma was less than the controls but this difference was not significant (Table 2). Elevated TIMP-1 has been found to be associated to high body weight [19, 20] though BMI of our patients was significantly lower than the control individuals; also BMI was not found to correlate with serum TIMP-1 level [20]. There are few studies which reported that the normal stoichiometric ratio of MMP-9 to TIMP-1 might alter in favour of MMP-9 in the asthmatic airway [21]. It is not yet clear as to whether TIMP-1 level in patients of asthma remains unaltered or increased [7]. Higashimoto et al. [22] reported a significant decrease in the concentration of *TIMP-1* in asthmatics where as Hoshino et al. [23] found increased levels of MMP-9 as well as TIMP-1. However, Cataldo et al. [7] reported that TIMP-1 levels remained unaltered while there was an increase in MMP-9 in asthmatics, thus shifting the balance in favor of protease (MMP-9). The exhaled breath condensate was analyzed for TIMP-1 in children with bronchiectasis by Karakoc et al. [24] and they reported no statistically significant difference in TIMP-1 levels in patients and healthy controls. The results obtained in the current study suggested that serum levels of TIMP-1 may not be a very good marker for the diagnosis of asthma, at least in the Indian population. 

We observed an increase in the level of serum cytokines in asthma patients as compared to the controls (Figure 1). The levels of cytokines suggested a switch from Type-1 to Type-2 cytokine predominance that may result in enhanced synthesis of IgE. The IL-1*β* has also been shown to induce aryl hydrocarbon receptor in murine model of asthma, in a dose and time-dependent manner [25]. It could switch on the transcription of IL-8 gene and thus leads to increased release of IL-8 from the lung cell cocultures [26]. The broncho alveolar lavage (BAL) fluid from smokers had been reported to contain higher levels of IL-1*β* than BAL fluid from nonsmokers, and the levels of IL-1 *β* had been related to the smokers' lung function [27]. In our study also there was a rise in the level of IL-1*β* in patients as compared to controls (*P* = 0.001). The IL-4 is a cytokine associated with Th2 type of immune response in the host. The role of IL-4 has been described in IgE production, and the activation of mast cells and eosinophils [28]. We observed the increase in IL-4 levels to be significant in patients of asthma in comparison to controls. Ohashi et al. analyzed patients with allergic rhinitis for serum IL-4 and reported the concentration of IL-4 to be significantly elevated [29]. They also reported a correlation between IgE and IL-4 levels in the sera of patients. Thus our results are in accordance with the previous reports suggesting the role of IL-4 in the production of IgE antibodies in the host. IL-5 has been a key mediator in eosinophil activation and its concentration in our controls was significantly lower (*P* = 0.001) than in the patients. Increased IL-5 mRNA expression and IL-5 protein levels have been reported to rise in BAL, sputum, and serum of asthmatics [30–32]. IL-5 has been reported to cause increased eosinophilia [33]. It is evident from the results of the present study and those reported in the literature that IL-5 plays a role in asthma by interacting with other inflammatory mediators. IL-6 has been a proinflammatory cytokine that has been reported to stimulate immune response mediated tissue damage leading to inflammation. Our results were in unison with the earlier reports, and the role of IL-6 in influencing the other interleukins (IL-1 *β* and IL-8) was also evident. It was observed in the present study that the concentration of IL-8 in the serum of patients of asthma increased significantly in comparison to control subjects. IL-8 was shown to regulate the chemotactic migration of neutrophils and has been an important molecule of inflammatory pathways. We have found more than fourfold increase in IL-8 concentration in patients which was found to be highly significant (*P* = 0.001), indicating the role of neutrophils in inflammation in asthma (Figure 1). These observations emphasized the fact that there is a complex series of inflammatory events in this disorder where eosinophils and neutrophils play interactive roles. IL-10 has been an anti-inflammatory cytokine and has pleiotropic effects in immunoregulation and inflammation. In addition, IL-10 has been known to inhibit the lymphokine production by Th1 but not Th2 clones and downregulate the Th1 cell differentiation [34, 35]. The results of the present study showed that the levels of IL-10 in patients of asthma increased significantly (*P* = 0.001) in comparison to controls. There have been conflicting reports in literature on the levels of IL-10 in asthma patients. Takaashi et al. in their study on Japanese subjects reported reduction in IL-10 level in sputum of bronchial asthma and in normal smokers as compared to healthy nonsmokers [36]. In a study carried out by Ceyhan et al. IL-10 in sera and induced sputum of asthma patients were found to be unaltered [37]. We found the IL-10 to be increased in patients of asthma. The elevated level of IL-10 in the serum of asthma subjects indicated an increase in Type-2 activity through which the production of IL-4 and IL-13 may promote an isotype switch to IgE. Thus, a prominent shift in patient cytokine milieu from Type-1 to Type-2 may have resulted in the elevated levels of total IgE which is also demonstrated in our patients.

 The PIM3 allele of **α**_1_ *AT* has been reported to be associated with the prevalence of COPD and decreased STIC [10, 11, 13]. We could not establish any association of this SNP (PIM3) with asthma but observed a novel polymorphism, Glu363Lys in exon-V of **α**_1_ *AT* upstream to PIM3, to be associated with bronchial asthma. Therefore, genotype Glu363Lys of **α**_1_ *AT* could be considered as an important genetic marker in asthma predisposition.

 The genotyping for *TIMP-1* was performed separately for male and female subjects, as this has been a sex linked gene. Hinterseher et al. analysed the coding region and selected parts of the promoter of *TIMP-1 *gene to study its polymorphic status with respect to aneurysm in Caucasians [38]. They identified and analyzed three exonic SNPs, C/T (rs1043428), T/C (rs4898), and a novel 344C/T, but failed to show any association between these SNPs and abdominal aortic aneurysm in Caucasian patients. However, a silent SNP Phe124Phe of *TIMP-1 *gene had been reported to be associated with abdominal aortic aneurysm in women [39]. The results of a study conducted on Australian women showed that a synonymous SNP Ile158Ile of *TIMP-1 *gene was associated with the prevalence of asthma [40]. The SNPs Phe124Phe and Ile158Ile of TIMP-1 gene had been earlier studied by us in COPD [11] but were not associated with the disease but in this paper SNP Phe124Phe of TIMP-1 has been found to be associated with asthma in Indian population. However, van Diemen et al. reported that the SNPs Phe124Phe and Ile158Ile were associated to decline in FEV_1_ in male Caucasian COPD subjects [41]. The pattern of allelic distribution of Phe124Phe in our subjects indicated strong association of this SNP with the prevalence of asthma in Indian population. It is a synonymous mutation unlikely to have a functional or structural effect on the protein, however, could be considered as marker for genetic association with asthma.

We explored the SNPs rs2070584 and rs6609534 of *TIMP-1 *particularly because they were found to be located in 3′ UTR of the *TIMP-1 *gene, which has been reported to have important role in the gene regulation via siRNA interaction(s). But we failed to find any association of both of these SNPs with asthma. However, a novel intronic SNP G3774A was found to be strongly associated with the occurrence of asthma; therefore, it could be considered as an important genetic marker.

**Figure 4:**
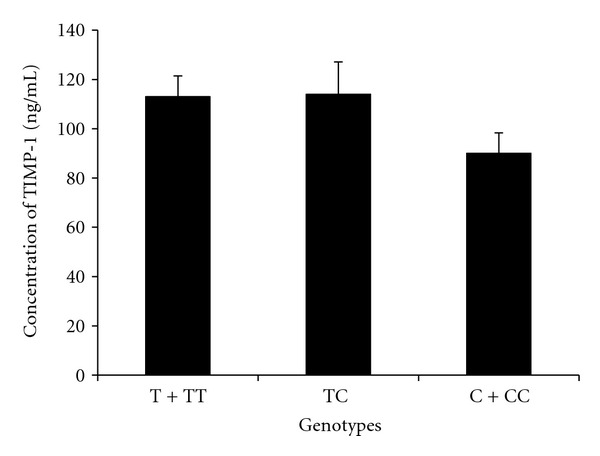
Concentration of TIMP-1 (mean ± SE) in the serum of male and female patients of asthma having different genotypes of SNP Phe124Phe of *TIMP-1* gene. TT (female) and T (male) are wild-type genotypes. TC, CC (female), and C (male) are mutant genotypes of *TIMP-1* gene.

The genotyping studies further demonstrated that SNP Glu363Lys of **α**_1_ *AT* gene was found to be associated with asthma. Although asthmatics had significantly lower STIC, this polymorphism did not show any correlation with *α*_1_AT level in sera of asthmatics, as the patients having all three genotypes GG, GA, and AA had almost similar levels of *α*_1_AT (Figure 2). The serum concentration of TIMP-1 in asthmatics with different genotypes of the novel polymorphism G3774A was not found to be significantly different from each other (Figure 3). The exonic SNP Phe124Phe in *TIMP-1 *was found to be associated with the prevalence of asthma in our study. The TIMP-1 serum concentration in asthma patients with different genotypes (T, TT, TC, C, and CC) was found to be similar to each other (Figure 4); as a result no association could be established between the antiprotease concentration and this silent exonic SNP.

 In conclusion the novel SNPs, Glu363Lys of **α**_1_ *AT* and 3774 (G to A) of *TIMP-1* gene showed strong association with asthma. In view of these observations we considered that Glu363Lys of **α**_1_ *AT* and G3774A and rs4898 of *TIMP-1* gene could be important genetic markers for use in better management of asthma. Our findings also suggested that the increased synthesis of inflammatory mediators and decreased expression of antiproteases under given genotypic background in patients must have contributed to the development of asthma.

## Figures and Tables

**Table 1:** List of SNapshot primers used for identification of SNPs of **α*_1_AT* and *TIMP-1 *genes.

Polymorphism	Gene	Primer sequence
Val213Ala	*α*_1_ *AT *	5′CTTCCACGTGGACCAGG3′
Glu363Lys	*α*_1_ *AT *	5′GGTTTGTTGAACTTGACCT3′
Glu376Asp	*α*_1_ *AT *	5′GTCTTCTTAATGATTGA3′
Novel 3770	*TIMP-1 *	5′CCCAATTTCAGTCTATCA3′
rs5953060	*TIMP-1 *	5′CAGAAGGCCGGGCCTTTG3′
rs4898	*TIMP-1 *	5′CATCACTACCTGCAGTTT3′
rs6609533	*TIMP-1 *	5′GTCCAATACCGTGTGATC3′
rs11551797	*TIMP-1 *	5′GTTTCCCTGTTTATCCAT3′
rs2070584	*TIMP-1 *	5′GCTGACAAATCACTGCCT3′
rs6609534	*TIMP-1 *	5′GATGCCCCATCCAAACAC3′

**Table 2:** Demographic and biochemical parameters of asthma patients and control subjects.

Parameters	Asthma	Controls	*P value
	(*n* = 202)	(*n* = 204)	
Age (mean ± SE) years	39.70 ± 1.12	33.00 ± 0.64	0.001
Sex (male/female)	93/109	126/78	—
Smoking index (mean ± SE)	26.27 ± 6.52	47.57 ± 8.03	0.001
Body mass index (mean ± SE)	22.87 ± 0.33	23.96 ± 0.25	0.009
Total IgE (mean ± SE) IU/mL	862.82 ± 52.80	186.50 ± 19.83	0.0001
STIC (mean ± SE) mg trypsin inhibited/mL of sample	0.98 ± 0.03	1.26 ± 0.05	0.001
TIMP-1 (mean ± SE) ng/mL	104.80 ± 5.47	111.14 ± 4.99	0.27

*P values were obtained by “*t*-test” analysis using SPSS 12 software.

**Table 3:** Distribution of genotype frequencies of SNPs of *α*_1_ *AT* and* TIMP-1* genes in asthma patients and control group.

SNP	Patients	Control	*χ* ^2^	*P value	OR (95% CI)^†^
Allele	(*n* = 202)	(*n* = 204)			
Val213Ala (*α*_1_ *AT*)					
(rs6647)					
TT	152 (75.24%)	167 (81.80%)			1
TC	46 (22.77%)	34 (16.67%)	2. 48	0.12	1.21 (0.97–1.51)
CC	4 (1.98%)	3 (1.40%)	0.25	0.62	1.20 (0.63–2.30)

Glu363Lys (*α*_1_ *AT*)					
(Novel)					
GG	135 (66.83%)	171 (83.80%)			1
GA	51 (25.24%)	26 (12.74%)	12.05	0.001	1.50 (1.23–1.84)
AA	16 (7.92%)	7 (3.43%)	5.58	0.02	1.58 (1.17–2.13)

Glu376Asp (*α*_1_ *AT*)					
(rs1303)					
AA	127 (62.87%)	140 (68.63%)			1
AC	54 (26.73%)	51 (25.00 %)	0.45	0.50	1.08 (0.86–1.35)
CC	21 (10.39%)	13 (6.37%)	2.43	0.12	1.29 (0.97–1.74)

rs5953060 (*TIMP-1*)					
CC	33 (30.27%)	26 (33.33%)			1
CG	43 (39.45%)	31 (39.74 %)	0.06	0.80	1.04 (0.77–1.39)
GG	33 (30.27%)	21 (26.92%)	0.31	0.58	1.09 (0.80–1.49)
C	48 (51.61%)	71 (56.34%)			
G	45 (48.38%)	55 (43.65%)	0.48	0.49	0.67 (0.43–1.07)

Base 3774 (*TIMP-1*)					
GG	80 (73.39%)	74 (94.87%)			1
GA	23 (21.10%)	3 (3.84%)	12.12	0.001	1.70 (1.39–2.09)
AA	6 (5.50%)	1 (1.28%)	3.07	0.07	1.65 (1.18–2.32)
G	82 (88.17%)	120 (95.23%)			
A	11 (11.83%)	6 (4.76%)	3.73	0.05	1.59 (1.08–2.35)

rs4898 Phe124Phe (*TIMP-1*)					
TT	37 (33.94%)	40 (51.28%)			1
TC	42 (38.53%)	29 (37.17%)	1.83	0.18	1.23 (0.91–1.66)
CC	30 (27.52%)	9 (11.53%)	8.84	0.002	1.60 (1.19–2.14)
T	49 (52.69%)	84 (63.49%)			
C	44 (47.31%)	42 (33.33%)	4.57	0.03	1.39 (1.03–1.89)

rs6609533 (*TIMP-1*)					
GG	46 (42.20%)	39 (50.00%)			1
GA	35 (32.11%)	26 (33.33%)	0.15	0.69	1.06 (0.79–1.42)
AA	28 (25.69%)	13 (16.67%)	2.29	0.13	1.26 (0.95–1.68)
G	61 (65.60%)	85 (67.46%)			
A	32 (34.40%)	41 (32.53%)	0.08	0.77	1.05 (0.76–1.45)

rs11551797 Ile158Ile (*TIMP-1*)					
CC	107 (98.16%)	74 (94.87%)			1
CT	2 (1.83%)	3 (3.84%)	0.73	0.39	0.68 (0.23–1.99)
TT	0 (0.00%)	1 (1.36%)			N/A
C	91 (97.84%)	121 (96.03%)			
T	2 (2.15%)	5 (3.96%)	0.57	0.45	0.67 (0.20–2.17)

rs2070584 (*TIMP-1*)					
GG	33 (30.27%)	33 (42.30%)			1
GT	46 (42.20%)	27 (34.61%)	2.39	0.12	1.26 (0.94–1.69)
TT	30 (27.52%)	18 (23.07%)	1.76	0.19	1.35 (0.86–2.12)
G	48 (51.61%)	78 (61.90%)			
T	45 (48.38%)	48 (38.09%)	2.32	0.13	1.27 (0.94–1.73)

rs2070534 (*TIMP-1*)					
AA	34 (31.19%)	27 (34.61%)			1
AG	47 (43.12%)	34 (43.58%)	2.64	0.10	1.31 (0.94–1.84)
GG	28 (25.69%)	17 (21.79%)	3.35	0.07	1.41 (0.98–2.02)
A	50 (53.76%)	81 (64.28%)			
G	43 (46.23%)	45 (35.71%)	2.47	0.12	1.28 (0.94–1.74)

*P values were obtained after adjusting it with age, BMI, and SI by regression analysis using SPSS 12 software.

^†^OR (95% CI): odds ratio (95% confidence interval). Genotype frequencies of male and female individuals for *TIMP-1* are given separately, since only single allele exists in male individuals.

**Table 4:** Distribution of allele frequencies of SNPs of *α*_1_ *AT* and *TIMP-1* genes in asthma patients and control group.

SNP	Patients	Control	*χ* ^2^	*P value	OR (95% CI)^†^
Allele					
Val213Ala (*α*_1_ *AT*)					
T	350 (86.63%)	368 (90.19%)			
C	54 (13.36%)	40 (9.80%)	0.44	0.51	1.15 (0.78–1.70)

Glu363Lys (*α*_1_ *AT*)					
G	321 (79.45%)	368 (90.19%)			
A	83 (20.54%)	40 (9.80%)	17.78	0.001	2.98 (1.79–4.96)

Glu376Asp (*α*_1_ *AT*)					
A	308 (76.23%)	331 (81.12%)			
C	96 (23.76%)	77 (18.87%)	0.74	0.39	1.15 (0.85–1.57)

rs5953060 (*TIMP-1*)					
C	157 (50.48%)	154 (54.61%)			
G	154 (49.52%)	128 (45.39%)	0.50	0.48	1.11 (0.84–1.46)

3774 (*TIMP-1*)					
G	265 (85.21%)	271 (96.09%)			
A	46 (14.79%)	11 (3.91%)	7.04	0.008	1.68 (0.27–2.22)

rs4898 Phe124Phe (*TIMP-1*)					
T	165 (53.05%)	192 (68.08%)			
C	146 (46.95%)	90 (31.21%)	5.38	0.02	1.39 (1.06–1.82)

rs6609533 (*TIMP-1*)					
G	188 (60.45%)	189 (67.02%)			
A	123 (39.55%)	93 (32.98%)	1.06	0.30	1.16 (0.88–1.53)

rs11551797 Ile158Ile (*TIMP-1*)					
C	307 (98.71%)	272 (96.45%)			
T	4 (1.29%)	10 (3.55%)	1.85	0.17	0.39 (0.07–2.28)

rs2070584 (*TIMP-1*)					
G	160 (51.45%)	171 (60.64%)			
T	151 (48.55%)	111 (39.36%)	2.03	0.15	1.22 (0.93–1.61)

rs2070534 (*TIMP-1*)					
A	165 (53.05%)	169 (59.93%)			
G	146 (46.95%)	113 (40.07%)	0.99	0.32	1.15 (0.87–1.52)

*P values were obtained after adjusting it with age, BMI, and SI by regression analysis using SPSS 12 software. ^†^OR (95% CI): odds ratio (95% confidence interval).
